# Concentrations of Particulate Matter and PM-Bound Polycyclic Aromatic Hydrocarbons Released during Combustion of Various Types of Materials and Possible Toxicological Potential of the Emissions: The Results of Preliminary Studies

**DOI:** 10.3390/ijerph17093202

**Published:** 2020-05-05

**Authors:** Karolina Bralewska, Joanna Rakowska

**Affiliations:** The Main School of Fire Service, Safety Engineering Institute, Slowackiego Street, 52/54, 01-629 Warsaw, Poland; jrakowska@sgsp.edu.pl

**Keywords:** particulate matter, polycyclic aromatic hydrocarbons, combustion products, fire smoke

## Abstract

The aim of this study was to compare the concentrations of particulate matter (PM) and PM-bound polycyclic aromatic hydrocarbons (PAHs) during the combustion of various types of materials (i.e., oak, beech, and pine wood, polypropylene, polyurethane, paper, cotton, and oriented strand board (OSB)), and to compare the carcinogenic, mutagenic, and toxic potential of the emissions during the burning of these materials. Personal portable sampling devices were used to collect samples and to determine concentrations of PM_4_, total suspended particles (TSPs), PM-bound PAHs. The samples were collected during controlled fires under laboratory conditions. The highest PM concentration was recorded during the burning of polyurethane (PM_4_-1818 mg/m^3^, TSP-2800 mg/m^3^), while the highest concentration of PAH mixture was recorded when burning OSB (628.5 µg/m^3^ PM_4_-bound; 791.2 µg/m^3^ TSP-bound PAHs). Thus, the highest carcinogenic (85.5 µg/m^3^), mutagenic (68.2 µg/m^3^) and toxic equivalents (26.4 ng/m^3^) of the PAH mixture were noted during OSB combustion. Carcinogenic potential (CP) of PAH group was determined mainly by phenanthrene (CP on average 21.6%) and pyrene concentrations (13.3%). The results of the study express possible adverse effects from PM-bound PAHs released during combustion for firefighters and other people staying near a fire site.

## 1. Introduction

Smoke from burning materials contains a wide variety of harmful gases (carbon dioxide (CO_2_), carbon monoxide, methane (CH_4_), nitrous oxide (N_2_O), nitrogen oxides (NO_x_), hydrogen cyanide (HCN), nonmethane volatile organic carbon (NMVOC)), polycyclic aromatic hydrocarbons (PAHs), and fine and coarse particulate matter (PM) [1,2,3,4,5,6,7]. The types and amounts of toxic chemicals produced and released during fires depend, among others, on the type of material burned [1]. Moreover, the composition of fire smoke also depends on the fire development phase and conditions (temperature, oxygen access). As the fire progresses, the production of all incomplete combustion products increases, usually 10–50 times [8]. To estimate particulate matter emissions from fire of various materials, emission factors (EFs) expressing the mass of PM emitted from 1 kg of fuel (g/kg or mg/kg) are used. EF depends on the fire, type of fuel, atmospheric conditions including wind direction and strength, or ventilation intensity. Initially, the emission factor of PM decreases, but in the next stage of a fire it begins to increase due to oxygen deficiency in the combustion area [9,10]. Therefore, it is very important to indicate the fire stage during which the emission assessment was carried out. Influence of the type of fuel and the combustion conditions on the amount of generated particulate matter have been shown in studies [9,11,12,13].

Particulate matter can be described by its “aerodynamic equivalent diameter” (AED). Particles of the same AED will tend to have the same settling velocity. The size of dust particles released during fire, similar to atmospheric air, ranges from nanometers to millimeters [14,15]. Due to the different ability of PM particles to move, different deposition of particles in the human respiratory tract and the characteristics of the most commonly used measuring devices for PM, it is customary for PM to be divided into fine (PM_2.5_), respirable (PM_4_), coarse (PM_10_), and total suspended particles (TSP) [14]. The particle size having 50% penetration for the respirable fractions is 4.0 μm [14,15]. These criteria have been developed specifically for the workplace atmospheres, and the occupational exposure data from PM are characterized based on an AED less than 4 μm [16,17]. Respirable fractions of PM that enter into the respiratory tract can caused serious health problems [18].

Polycyclic aromatic hydrocarbons (PAHs) are formed during incomplete combustion of organic materials and can occur both in the particle and gas phase. PAHs are generated from either natural combustion sources, such as wildlands, or municipal structural fires. Of the 17 PAHs released during fires, the International Agency for Research on Cancer (IARC) classified benzo[a]pyrene (BaP) as carcinogenic to humans (Group 1) and thus as a carcinogenic marker. Several other PAHs have been classified as probably carcinogenic to humans (Group 2A or 2B) [19]. It has been proven that PAHs can cause also mutagenic, teratogenic, and immune-suppressing effects [20,21]. According to epidemiological studies, a measure of the toxicological potential of a PAH mixture is carcinogenic potential (CP) of the individual compounds of the PAH mixture [22] and carcinogenicity (CEQ), mutagenicity (MEQ), toxicity (TEQ) equivalents relative to the carcinogenicity or mutagenicity of BaP, or to the toxicity of 2,3,7,8-tetrachlorodibenzo-p-dioxin, respectively [23,24,25].

Although research on the concentrations of pollutants released during fires have been the subject of many studies since the 1980s, there is still a need to recognize the hazards associated with fires and emissions of combustion products and a way to assess toxicological potential of emission. Measurements of the concentration of combustion products were carried out by [1,26,27,28,29] in real structural, industrial, and wildland fire or in laboratory scale. They consisted of different burning fuel, oxygen access, energy supply, and the presence of free radicals. Yields of toxic combustion products from burning materials vary considerably between different stages and types of fire and ventilation conditions. It is important that any laboratory studies of such phenomena be carried out as close as possible to real fire conditions. Laboratory tests are designed as specific cases of real fires. All these studies show that it is difficult to make measurements of real conditions without interfering with the environment.

The article presents an innovative method of fire simulation in laboratory conditions. The aim of this study was to determine the concentrations of PM and PM-bound PAHs during fire and assessment of the carcinogenic, mutagenic, and toxic potential of PM-bound PAH mixture released during fire. The novelty of this manuscript and described experiment relies on finding possible dangers for firefighters resulting from combustion of different materials used in domestic and municipal buildings and comparison of how these materials differ regarding the amount and composition of the corresponding PM emissions and what material burns faster or more violently. An overview of the main sources of particle emission to atmosphere during fires has been presented. As the fuel, eight types of materials were tested. The emissions factors of biomass sampled in an open environment were also considered.

## 2. Materials and Methods

### 2.1. PM and PAH Analysis

**Figure 1:**
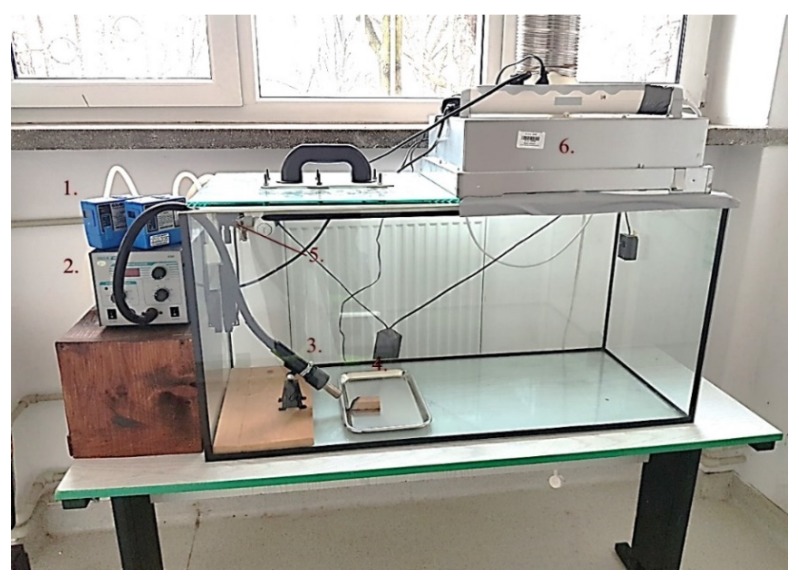
Measuring stand: 1—GilAir-3 personal aspirators; 2—device regulating heat release; 3—source of radiation; 4—tray with tested combustible material; 5—heads for collecting respirable and total suspended particle (TSP) fractions; 6—exhaust smoke.

The study consisted of simultaneously collecting a respirable fraction (PM_4_) and total suspended particles (TSPs) released during the combustion of materials such as beech, oak, and pine wood (blocks measuring 10 cm × 10 cm × 2 cm), paper (book), cotton (100% cotton jersey), polypropylene, polyurethane, and oriented strand board (OSB) (pieces measuring 20 cm × 20 cm × 5 cm). The measurements were carried out during controlled fires under laboratory conditions, for each material separately. The measuring stand consisted of a glass, heat-resistant combustion chamber measuring 1.2 m × 0.6 m × 0.6 m with a volume of 0.36 m^3^, covered with a heat-resistant glass cover with an opening of 50 cm^2^ (for placing aspirator heads); ignition nozzle with adjustable temperature; and a ventilation system enabling the discharge of volatile combustion products (Figure 1). The samples were burned at 400 °C. The content of PM_4_ and TSP fractions was determined by the gravimetric method [30]. The collection of samples was carried out using two GilAir-3 personal aspirators (certified in accordance with ISO 13137-2013 standard) [31] and two types of heads: cyclone for the respirable fraction and a head for collecting TSP. The air flow rate was 2.2 L/min for PM_4_ and 2.0 L/min for TSP. Due to the technical capabilities of the devices, each measurement lasted 10 min. With a longer intake time, the filters clogged, and the devices turned off arbitrarily. The particles of both fractions were collected on glass filters (GF2-025, ø 25 mm, CAT no GF2-025, CHMLAB Group), which were conditioned in the weighing room (air humidity 45% ± 5%, air temperature 20 ± 2 °C) for 48 h before each weighing. To determine the filter mass, a microbalance weight with a deionizing gate (MYA 5.3Y.F, RADWAG, Radom, Poland, resolution of 1 μg) was used. The filters with PM_4_ and TSP samples were subjected to the same conditioning and weighing procedures.

Details of the polycyclic aromatic hydrocarbons (PAHs) analysis can be found in [32]. Briefly, two-stage extraction was performed to isolate PAHs from PM_4_ and TSP samples. Each sample of PM_4_, TSP, and blank samples (pure glass fiber filters) were placed in a glass vial containing 10 mL of dichloromethane (DCM) and kept in an ultrasonic bath for 30 min. The extract was then filtered through membrane filters (PTFE 45 µm), and again placed in a 10 mL of DCM for ultrasonic extraction. Then, 3 mL acetonitrile was added, and the extract was again concentrated to 1 mL in a nitrogen stream. The PAH contents on the prepared extract was determined by high-performance liquid chromatography (HPLC), using a Surveyor Finnigan chromatograph from Thermo Scientific with a UV/Vis detector (25 µL injection loop, C18 chromatographic column—Hypersil Green PAH 5 µm, 250 × 4.6 mm) under the control of ChromQuest software. The mobile phase (acetonitrile and water solution 60:40 *v*/*v*) flow rate was 1.2 cm^3^/min, and the wavelength was 254 nm. The concentrations of 15 compounds from PAH mixture were determined: naphthalene (Na), acenaphthalene (Ace), fluorene (Fl), phenanthrene (Phe), anthracene (Ant), chrysene (Ch), fluoranthene (F), pyrene (Py), benzo[a]anthracene (BaA), benzo[b]fluoranthene (BbF), benzo[k]fluoranthene (BkF), benzo[a]pyrene (BaP), dibenzo[a,h]anthracene (DahA), benzo[g,h,i]perylene (BghiP), and indeno[1,2,3-cd]pyrene (IcdP). The calibration curve was plotted for each PAH separately using a standard mixture of 15 PAHs (610 PAH Calibration Mix A, RESTEK) containing each compound at a known concentration. The limit of detection for each PAH was 0.5 ng/mL. Recovery rates were from 81.94% (Na) to 92.24% (BghiP) and the standard deviation was from 7.2% (BbF) to 22.6% (Ch).

All tests were performed seven times for each type of material. In order to get a statistical representation of PM mass on filters, 112 samples were analyzed (14 for each burned material, including seven PM_4_ and TSP samples). According to the gravimetric analysis, the standard deviations obtained from the three times weighing masses of filters both before and after sampling were in this study all less than 0.0005 mg. Pearson correlation coefficient was used to compare differences in means of the PM fractions and relative PAHs content in this fractions across the eight burned material types. Differences were considered statistically significant when *p* < 0.05. Pearson correlation coefficients (*r*) and statistical significance (*p*) values are given below the charts.

### 2.2. Assessment of Carcinogenic, Mutagenic, and Toxic Potential of PM-Bound PAH Compounds Released during Combustion

The assessment of carcinogenic, mutagenic, and toxic potential of PM-bound PAH compounds released during combustion of various types of materials was made by using equivalents: carcinogenic (CEQ), mutagenic (MEQ), and TCDD-toxic (TEQ) [23]. For each burned material, CEQ (1), MEQ (2), TEQ (3) were computed by multiplying the concentrations of selected compounds from the PAH group by the corresponding toxicity equivalence factors (TEF), mutagenic equivalence factors (MMC—minimum mutagenic concentration) and carcinogenic equivalence factors (TCDD-TEF) [24,25]. The values of TEF, MMC, and TCDD-TEF factors for the particular PAH, used in calculations were taken from [23,24]. From Equation (4), the contribution of individual compounds of the PM-bound PAHs to the total carcinogenic potential (CP) of PAH mixture was calculated [22].

CEQ = 0.001 × ([Na] + [Acy] + [Ace] + [Fl] + [Phe] + [F] + [Py]) + 0.01 × ([Ant] + [Ch] + [BghiP]) + 0.1 × ([BaA] + [BbF] + [BkF] + [IcdP]) + 1 × [BaP] + 1 × [DahA] (1)

MEQ = 0.00056 × [Acy] + 0.082 × [BaA] + 0.017 × [Ch] + 0.25 × [BbF] + 0.11 × [BkF] + 1 ×[BaP] + 0.31 × [IcdP] + 0.29 × [DahA] + 0.19 × [BghiP] (2)

TEQ = 0.000025 × [BaA] + 0.00020 × [Ch] + 0.000354 × [BaP] + 0.00110 × [IP] + 0.00203 × [DahA] + 0.00253 × [BbF] + 0.00487 × [BkF] (3)

CP_i_ = [(PAH*_i_*/BaP × TEF*_i_*)/∑PAH*_i_*/BaP × TEF*_i_*] × 100% (4)

## 3. Results

### 3.1. PM Concentrations

**Figure 2:**
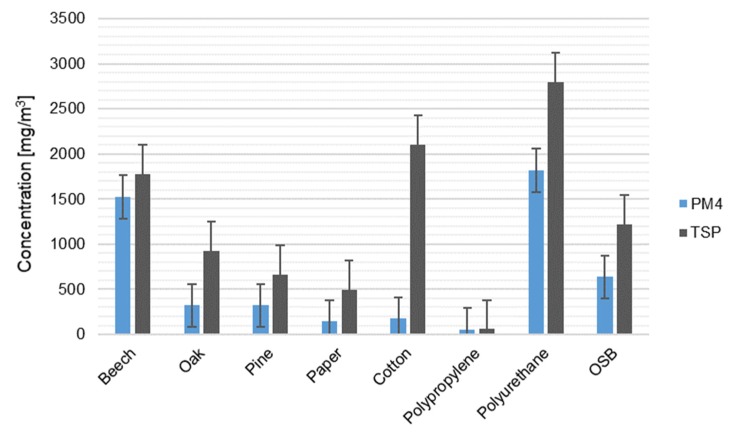
Concentrations (mg/m^3^) of respirable fraction of particulate matter (PM_4_) and total suspended particles (TSPs) during a 10-minute combustion of various types of materials (*r* = 0.757, *p* = 0.03).

The concentrations of two particulate matter fractions—a respirable fraction (PM_4_) and total suspended particles (TSPs)—present in fire smoke are presented in Figure 2. The concentrations of particulate matter released during the combustion of various materials differed significantly. The highest concentrations of particulate matter, both PM_4_ and TSP, occurred during combustion of polyurethane (PM_4_ 1818 mg/m^3^, TSP 2800 mg/m^3^). A high TSP concentration in relation to the other materials was also recorded when burning cotton (2100 mg/m^3^) and beech wood (1523 mg/m^3^). Assuming that PM_4_ is a certain part of the TSP [33,34] and assuming the reliability of the heads used for sampling, the experiment shows that during the combustion of cotton, mainly coarse PM with an aerodynamic diameter greater than 4 µm was emitted. It was similar in the case of paper burning. However, during combustion of polypropylene, beech wood, and polyurethane, particles with diameters smaller than 4 µm were mainly emitted. In the case of other materials, i.e., oak, pine wood, and OSB, the percentage of PM_4_ in total PM released during their combustion was close to 50%. The lowest concentration of PM was recorded during combustion of polypropylene (PM_4_ 54 mg/m^3^, TSP 60 mg/m^3^). Differences between PM concentrations accompanying the combustion of various materials depended primarily on their mass and volume, physical and chemical properties, including the density of the material being burned, the mechanism of heat transfer, and chemical reactivity [35]. The type of combustion (flaming and smoldering) and the rate of fuel mass loss over time were closely related to these properties [36]. Among the analyzed materials, only paper burned in flame, polypropylene melted, and no flames or smoke were observed during its combustion. It should be noted that during the combustion of polypropylene, the silicone tubes connecting the heads with the pumps stuck together, which probably understated the noted result. Beech, oak and pine wood, cotton, polyurethane, and OSB burned without flame with the release of large amounts of smoke. Therefore, the highest PM concentrations were recorded for flameless combustion, while they were lower for flame combustion. This was also confirmed by the research described in [2]. The highest emission of particulate matter occurred during the burning of high-density materials, i.e., beech and oak, OSB, polyurethane, and cotton [37,38]. According to the observations carried out during the experiment, cotton and polyurethane were the fastest to ignite and burn. It was influenced by the high thermal conductivity of these materials. Therefore, it caused a high concentration of PM during the combustion of these materials in the first stage of the fire [35]. The PM concentrations measured during the experiment, except for polypropylene combustion, are definitely higher than the PM concentrations recorded during experimental fires of pine forest in Siberia, Asia, where the average TSP concentration found for five fires in larch forest was 50 mg/m^3^ [39]. The measured concentrations are also several thousand times higher than the PM_2.5_ concentration in Georgia, USA, recorded during the burn seasons of 2015–2018 (8 µg/m^3^) [40]. The differences in concentrations may result from the duration of the test (averaging period), the fire stage that the tests included and the prevailing conditions, e.g., the oxygenation level of the fire, the size of the combustion area. In the research presented in this article, this time was short because it did not exceed 10 min. This was due to the technical limitations of aspirators and the rapid filling of filters. The tests were carried out with limited ventilation (under cover), which could also affect the type of combustion and the concentration of PM [9,10]. The relatively small volume of the combustion chamber in which the experiment was carried out was also likely to affect high PM concentrations. Nevertheless, it can be assumed that in the case of fires in larger spaces (e.g., outdoors), the concentration of PM will be lower than that during smaller fires (e.g., in apartments). These are the results of preliminary studies and the measurement method used has some limitations. However, the relationship of the level of pollutant emissions during a fire depending on the type of material burned has been confirmed.

**Figure 3:**
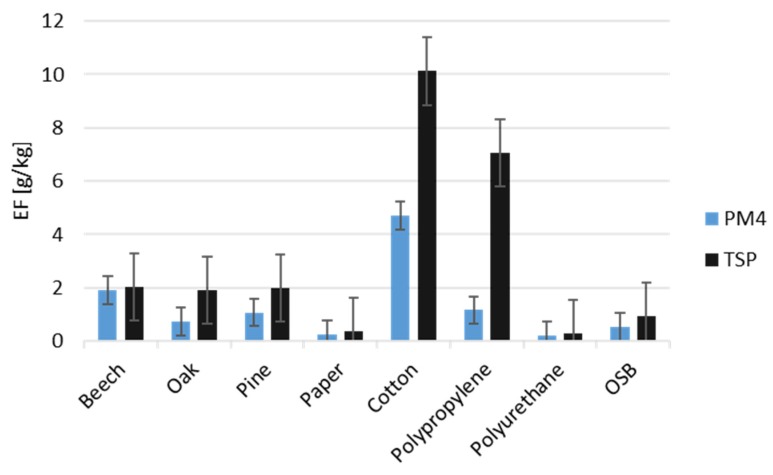
Emission factors during combustion of different types of materials (*r* = 0.849, *p* = 0.008).

According to Figure 3, the highest mass of PM emitted from 1 kg of fuel was created as a result of burning cotton (10 g/kg) and polypropylene (7 g/kg). The observations relate to the first stage of the fire, and this tendency may have changed with longer burning and thus sampling [10]. The amounts of PM generated during a fire of beech, oak, and pine wood, per kilogram of burnt material, were close to the value when agricultural biomass was burning [24]. In the event of large fires, both industrial and wildland, the resulting anthropogenic air pollution was identified as a factor causing human health problems in areas many kilometers away from the source of the emission [41,42]. Episodic, short-term exposure of people to airborne particulate matter at concentrations more than hundreds of times higher than ambient air quality is known to have adverse health impacts [18,34,40].

### 3.2. PAH Concentrations

**Figure 4:**
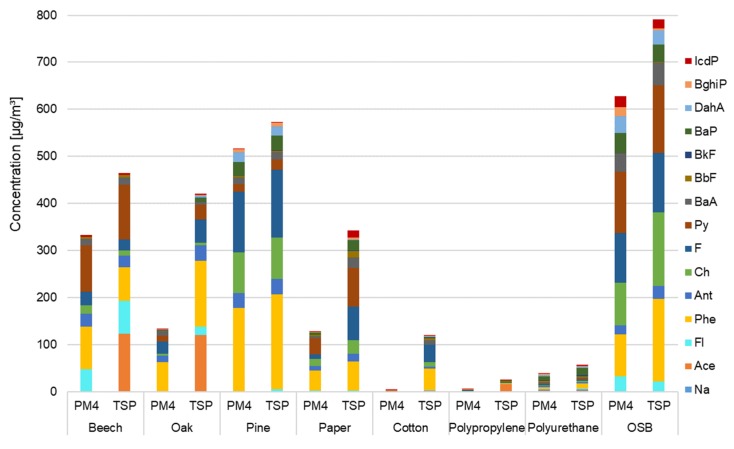
Concentration of 15 polycyclic aromatic hydrocarbons (PAHs) (µg/m^3^) related to respirable fraction (PM_4_) and total suspended particles (TSPs) during burning of various types of materials (*r* = 0.944, *p* < 0.001).

The total concentrations of the 15 PAH compounds (∑PAH) and concentrations of individual compounds from this group associated with respirable (PM_4_) and total suspended particles (TSP) released during the combustion of various types of materials are shown in Figure 4. Concentrations of both ∑PAH and individual compounds of the mixture differed depending on the material burned. ∑PAH associated with PM_4_ was 3.4–628.5 µg/m^3^, while ∑PAH associated with TSP was 25.1–791.2 µg/m^3^. The highest concentration of PM-bound PAHs was noted during combustion of oriented strand board (628.5 µg/m^3^ for PM_4_-bound PAHs and 791.2 µg/m^3^ for TSP-bound), while the lowest was during polypropylene combustion (5.0 µg/m^3^ for PM_4_-bound PAHs and 25.1 µg/m^3^ for TSP-bound). Burning cotton is also noteworthy. During the combustion of this material, the concentration of TSP-bound PAHs was about 35 times higher than the concentration of PM_4_-bound PAHs. Although ∑PAH during combustion of polypropylene and polyurethane was lower in comparison to other materials, it does not mean that they do not pose a threat. During fires of these materials, other gaseous hazardous substances may be released (e.g., carbon monoxide, hydrogen cyanide, hydrogen chloride) [1,2,3,4,5,6,7]. Low concentrations of PAHs during the combustion of polypropylene and polyurethane may have been associated with the technical problems of measuring devices because the combustion of polypropylene caused clogging of the tubes with the heads. TSP-bound PAHs released during the combustion of three types of wood were at a similar level, close to 500 µg/m^3^, with the highest PAH emission during the burning of coniferous wood and the lowest during combustion of oak wood (421–572 µg/m^3^). Still assuming that PM_4_ is part of TSP, during the combustion of OSB, polyurethane, pine, and beech, PAH compounds were mainly associated with respirable particles, while when burning oak, paper, cotton, and polypropylene, PAHs were mainly associated with coarse particles with aerodynamic diameters greater than 4 µm. The combustion materials whose concentrations of PM and PAHs were the highest do not coincide. Therefore, high particulate matter emissions when burning a given material do not mean high PAHs emissions and vice versa.

Among the 15 determined PM_4_-bound PAHs, phenanthrene had the highest concentrations during burning of oak, pine, and paper (42.4–176.8 µg/m^3^); pyrene during burning of beech wood (99.4 µg/m^3^) and OSB (130.8 µg/m^3^); benzo[g,h,i]perylene during burning of polypropylene (0.8 µg/m^3^); and benzo[a]pyrene during combustion of polyurethane (8.2 µg/m^3^). Among the 15 TSP-bound PAHs, acenaphthalene had the highest concentration during combustion of beech (122.9 µg/m^3^) and polypropylene (16.2 µg/m^3^); phenanthrene during combustion of oak (140.6 µg/m^3^), pine (201.1 µg/m^3^), OSB (175.4 µg/m^3^), and cotton (45.3 µg/m^3^); pyrene during paper combustion (82.9 µg/m^3^); and benzo[a]pyrene for polyurethane combustion (14.5 µg/m^3^). PM-bound PAHs’ concentration when burning 8 types of materials is lower than that during wind tunnel simulations of open burning for agricultural and forest biomass fuels including cereal grasses, agricultural tree pruning, and fir and pine wood (5–683 mg/m^3^) [6]. The PM-bound ∑PAH estimated during experiment is comparable, for example, to PAH concentrations measured directly during overhaul events by Baxter and his team [26]. Moreover, the masses of PAHs determined in total suspended particles during 10 min of OSB combustion (13.8 ng/g) and combustion of each of the wood species (pine 11.4 ng/g, beech 8.4 ng/g, oak 8.4 ng/g) are similar to the masses of PAH deposited in hoods during various stages of a fire in a furnished apartment: attack and overhaul (6.1 ng/g), search and overhaul (6.7 ng/g), attack and vent (4.5 ng/g), search, overhaul or vent (3.4 ng/g), and overhaul and attack (15.8 ng/g) [43]. The total sum of PAH concentrations during combustion of beech, oak, pine, paper, cotton, polypropylene, polyurethane, and OSB associated both with PM_4_ and TSP is higher than concentrations of PM_2.5_-bound PAHs in the breathing zone of firefighters at Portuguese fire stations [28] and higher than ∑PAH (both particulate-bound and gas-phase PAHs) measured during large boreal wildfire (May–August 2016) in central Canada (average ΣPAH 0.8 µg/m^3^, maximum daily average ΣPAH 2.8 µg/m^3^) [44]. The lowest concentration of PM_4_-bound PAHs recorded during cotton combustion is about 8 times higher than the total PAH concentration during the work shift in a Portuguese fire station. However, in the cited studies, PAH concentrations were monitored in firefighters that were not directly involved in firefighting activities. Due to different methods and sampling times, the above comparisons are for reference only. PAH concentrations in described research can be overstated due to the small volume of the combustion chamber. However, the order of magnitude [µg] suggests that they were still high compared to PAH concentrations in atmospheric air or in fire station rooms. This indicates the problem of high PAH concentrations directly at the fire site and in its first stage.

**Figure 5:**
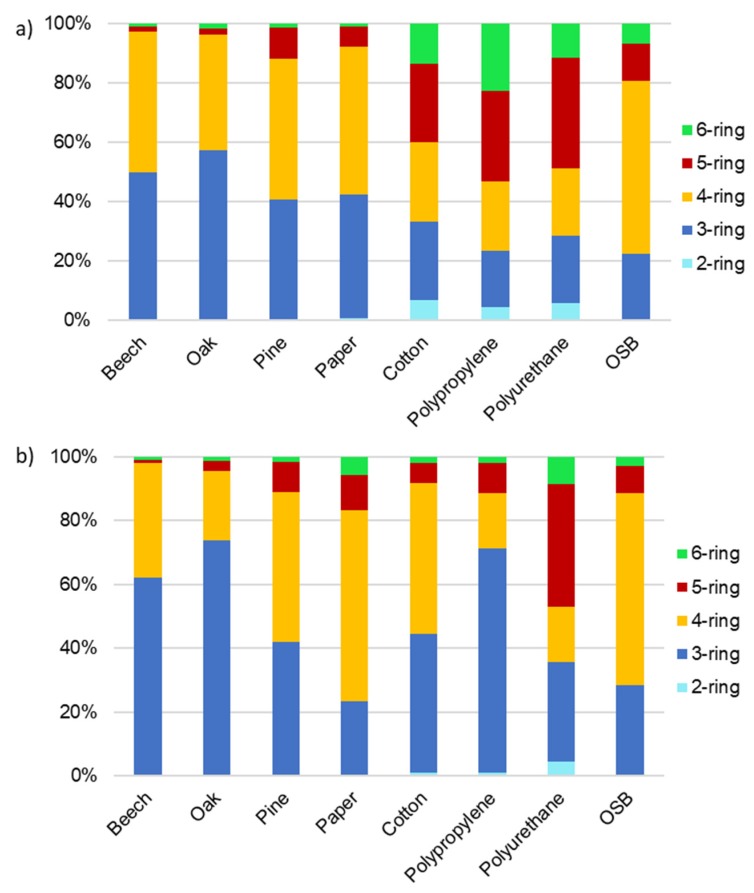
Profiles of PM_4_- (**a**) and TSP-bound (**b**) PAHs during burning of various types of materials (*r* = 0.919, *p* < 0.001).

PAHs can be present in both the particulate and gaseous phases, depending on their volatility. Light-molecular-weight PAHs with two or three aromatic rings are emitted in the gaseous phase, while high-molecular-weight PAHs with five or more rings are emitted in the particulate phase. Due to similar physicochemical properties of compounds with the same number of rings, PAH profiles (i.e., the percentages of individual PAH compounds in total PAH concentration) divided into 2-, 3-, 4-, 5-, and 6-ring compounds are presented in Figure 5. Not surprisingly, the contribution of 2-ring naphthalene in the total concentrations of PM_4_- and TSP-bound PAHs during combustion of most materials was insignificant (<1%). Naphthalene is a volatile compound with low molecular weight, and probably its concentration was much higher in a gaseous state [39]. Concentrations of this compound were slightly higher during combustion of cotton, polypropylene, and polyurethane, and it settled mainly on respirable particles. It had a 5% share in the sum of PAH concentrations related to the respirable fraction for the above materials. PAH profiles during the burning of three species of wood were similar. Compounds with 3 and 4 rings had the greatest shares in ΣPAH during wood burning (on average 94%) for all analyzed species and for both PM fractions. Five- and 6-ring PAHs had a low share when burning beech and oak (below 5%). During pine burning, the concentration of 5- and 6-ring PAH compounds accounted for about 12% of the total PAH concentration. It applies both PM_4_- and TSP-bound PAHs. The PM_4_-bound PAH profile for paper burning was similar to the PAH profile for wood burning. The burning of paper was also accompanied by the emission of mainly 3- and 4-ring PAHs. They constituted 92% and 83% of total concentration of PM_4_- and TSP-bound PAHs, respectively. The 5- and 6-ring PAHs had a higher share in ∑PAH during burning of cotton, polypropylene, and polyurethane. This was particularly visible in the PM_4_-bound PAH analysis (cotton 40%; polypropylene 55%; polyurethane 50%). In the case of PAHs related to total suspended particles, this share was a little lower (cotton 17%; polypropylene 30%; polyurethane 45%). Profiles of PAHs related to PM_4_ and TSP during OSB combustion were similar. OSB burning caused emissions of 3- and 4-ring compounds, which accounted for 80% and 88% of the total concentration of PM_4_- and TSP-bound PAHs, respectively.

The relative distribution of PM-bound PAH compounds was dominated by PAH compounds with 2–3 rings, while 5- and 6-ring PAH compounds contributed the most to the TEQ, MEQ, and CEQ values. Low-molecular-weight hydrocarbons in the gas phase act as precursors in the pyrosynthesis of PAH compounds that takes place at temperatures above 500 °C [45]. Therefore, our research will be continued at higher temperatures in the next stage.

### 3.3. Toxicological Potential of PM-Bound PAH-Compounds

Table 1 presents the values of carcinogenic (CEQ), mutagenic (MEQ), and toxic (TEQ) potential of PM-bound PAH compounds released during combustion of various types of materials. The carcinogenic potentials (CP) of the individual PAHs are presented in Figure 5. The highest CEQ, MEQ, and TEQ values occurred during OSB combustion. CEQ during the combustion of this material was about 140 times higher than that for cotton burning, while MEQ and TEQ were 136 and 88 times higher, respectively. High toxicological potential, relative to other materials, also occurred when burning pine. Although PAH concentrations during combustion of polyurethane were many times lower than those during combustion of beech, oak, paper, and cotton (Figure 3), CEQ and MEQ during burning of polyurethane were higher. In the case of real fires, such values may indicate possible adverse effects primarily to victims and witnesses of the fire, firefighters, police officers, rescuers, or onlookers.

CEQ estimated for all analyzed materials are several hundred times higher than CEQ calculated based on PAHs concentrations in the garage and changing room of selected fire station in Poland, where they were in the range of 21–82 ng/m^3^ [46].

**Figure 6:**
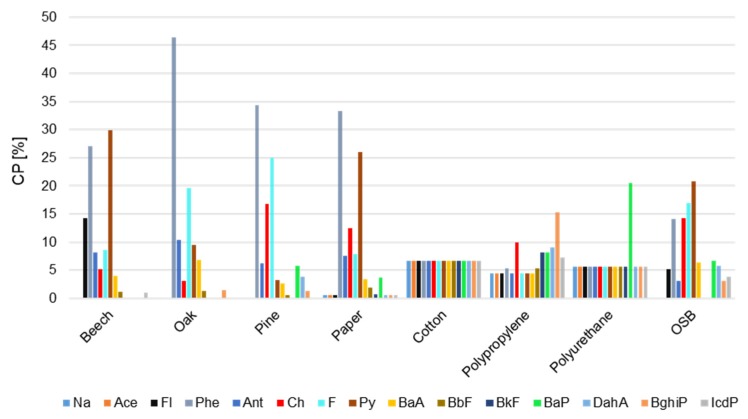
Carcinogenic potential (CP %) of individual compounds from the PM_4_-bound PAHs during burning of various types of materials.

**Figure A1:**
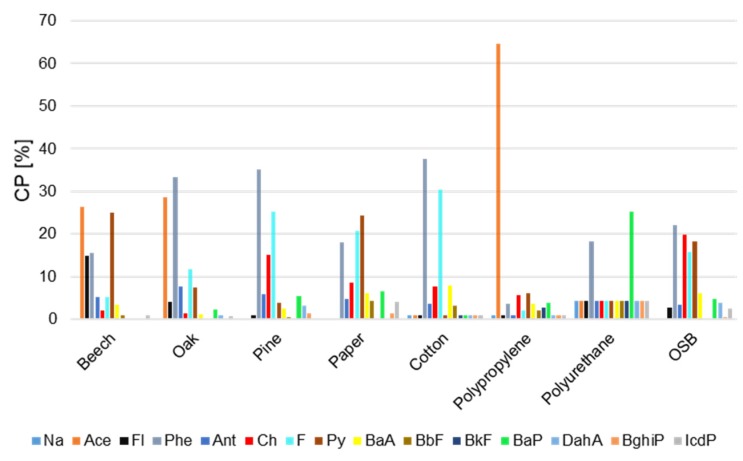
Carcinogenic potential (CP %) of individual compounds from the TSP-bound PAHs during burning of various types of materials.

Depending on the type of material burned, various PAH compounds had the largest contribution in carcinogenic potential of PAH mixture (Figure 6). The highest CP had Phe (33–46%) during burning of oak, pine, and paper. During burning of beech, carcinogenic potential was mainly determined by high concentrations of Py (30%) and Phe (27%). In the case of combustion of other materials, for OSB the highest CP had Py (21%), for polyurethane it was BaP (20%) and for polypropylene it was BghiP (15%). The same CP for most PAH compounds when burning cotton and polyurethane resulting from adjusting for these compounds’ concentrations corresponding to threshold values resulting from the sensitivity of the analytical method (0.5 ng/mL). The levels of detection for all PAHs were the same, and the estimated CP values for some compounds were equal. Carcinogenic potential (CP %) of individual compounds from the TSP-bound PAHs during burning of various types of materials are presented in Figure A1 (Appendix A).

## 4. Conclusions

Studies have shown that differences in the type of combustion of each material (smoldering, flameless, with flame) and the speed of ignition of each material have an impact on the amount of particulate matter emissions and PM-bound PAHs. The highest PM concentrations occurred during combustion of polyurethane, while the highest emission of PM-bound PAHs occurred during the combustion of oriented strand board. Among the 15 PM-bound PAHs, phenanthrene and pyrene had the highest concentrations in fire smoke. The carcinogenic (CEQ), mutagenic (MEQ), and toxic (TEQ) potential of PM_4_-bound PAH compounds released during combustion of beech, oak, pine wood, paper, cotton, polypropylene, polyurethane, and oriented strand board constitutes a possible adverse effects for people directly exposed to fire, including victims, firefighters, onlookers, and representatives of emergency services.

The conclusions from the investigations are as follows:

- The amount and type of hazardous substances present in fire smoke depend on the material that has been burned. Fires often involve objects/places where a combination of combustible materials occurs. The conducted tests are only the preliminary stage. They indicate the direction of further research and can be the basis and premise for research in more extensive forms, e.g., repeating an experiment with the same materials with longer sampling time and more repetitions, combining materials or using other materials, changing fire conditions (larger combustion chamber, use of other measuring equipment), measurement of the concentration of other combustion products, measurement of the concentration of other PM fractions (e.g., PM_1_, PM_2.5_, PM_10_).
- Although the measurements and calculations include only the concentrations of PM and PM-bound PAHs, the toxicological potential of emissions are high compared to other studies and may indicate high probability of adverse health effects during the combustion of all analyzed types of materials. Toxicological potential of emissions would be even higher if other PM-bound substances (e.g., metals, alkylated- and oxygenated-PAH compounds, and PAH-compounds containing heteroatoms) or gaseous pollutants released during fires were examined.
- Despite the fact that each fire is unique in terms of the type and amount of burning material and combustion conditions, additional studies are needed to better characterize the effects of fuel type and fire stage on exposure levels. Although smoke is a complex mixture and it was not possible to measure all of its components or potential health effects, we were able to demonstrate excessive or unhealthy exposures on the PM and PM-bound PAHs.

## Figures and Tables

**Table 1:** Mean values of the risk indicators (carcinogenic (CEQ), mutagenic (MEQ), and toxic (TEQ)) for PM-bound PAHs during burning of various types of materials.

Risk Indicator		Beech	Oak	Pine	Paper	Cotton	Polypropylene	Polyurethane	OSB
CEQ [µg/m^3^]	PM_4_	3.3	1.9	53.3	6.5	0.6	1.0	11.4	85.8
	TSP	3.7	15.0	53.7	28.8	4.3	1.5	18.1	76.4
MEQ [µg/m^3^]	PM_4_	3.8	2.0	40.6	6.5	0.5	0.9	11.0	68.2
	TSP	4.2	12.4	42.3	33.8	4.2	1.4	17.6	59.2
TEQ [ng/m^3^]	PM_4_	3.7	0.3	0.5	0.8	0.3	0.4	2.5	26.4
	TSP	4.6	3.8	0.4	15.7	1.4	0.3	2.8	21.2

OSB—oriented strand board.
